# Cognitive Impairment and Associated Factors Among Adult Hyperthyroid Patients at Bale Zone Hospitals, Southeast Ethiopia, 2024: Institution‐Based Cross‐Sectional Study

**DOI:** 10.1002/hsr2.72902

**Published:** 2026-07-30

**Authors:** Ephrem Tesfaye Mihretie, Girmaye Tesfaye, Yordanos Sintayehu, Ferhan Ahmed, Bereket Gezahegn, Niguse Serbecha, Zerihun Shiferaw, Awel Turki, Fikreab Desta

**Affiliations:** ^1^ Department of Medical Physiology, School of Biomedical Sciences, College of Medicine and Health Sciences Madda Walabu University Bale‐Robe Ethiopia; ^2^ Department of Anatomy, School of Biomedical Sciences, College of Medicine and Health Sciences Madda Walabu University Bale‐Robe Ethiopia; ^3^ Department of Public Health, College of Medicine and Health Sciences Madda Walabu University Bale‐Robe Ethiopia; ^4^ Department of Biochemistry, School of Biomedical Sciences, College of Medicine and Health Sciences Madda Walabu University Bale‐Robe Ethiopia

**Keywords:** cognitive impairment, Ethiopia, hyperthyroidism, MMSES, thyroid function

## Abstract

**Background and Aim:**

Cognitive impairment is an inclusive term used to describe the impairment of different domains of cognition. It is a growing public health concern and it can be influenced by hyperthyroidism. While the association between hyperthyroidism and cognitive impairment had been documented in other settings, evidence from Ethiopia remains limited. The aim of this study was to determine the prevalence of cognitive impairment and the associated factors among hyperthyroid patients attending Bale zone hospitals, Southeast Ethiopia.

**Method:**

An institution‐based cross‐sectional study was conducted from February to May 2024 among 406 hyperthyroid patients selected using a systematic random sampling technique. Cognitive impairment was assessed using the Mini Mental State Examination (MMSE) tool. Data were entered into EpiData Manager version 4.6.0.0 and exported to STATA version 17 for statistical analysis. The analysis began with descriptive statistics, followed by binary logistic regression. Variables with a *p* value < 0.25 in bivariable analysis were entered into multivariable binary logistic regression. The strength of association was determined by the adjusted odds ratio with a 95% confidence level.

**Result:**

The prevalence of cognitive impairment was 29.06% (95% CI: 24.84–33.68). The odds of cognitive impairment were higher in females, rural residents, and those with primary education. Older age (AOR = 1.09), higher free triiodothyronine levels (FT3) (AOR = 1.03), poor sleep quality (AOR = 2.24), and lower wealth (middle: AOR = 0.18; rich: AOR = 0.19) were linked to higher odds of cognitive impairment. Avoiding alcohol consumption (AOR = 0.16) helped lower the odds of cognitive impairment.

**Conclusion:**

Cognitive impairment is a significant health problem among hyperthyroid patients in southeast Ethiopia. Old age, higher FT3 levels, poor sleep quality, and low socioeconomic status are the key associated factors.

**Abbreviations**
AOR: Adjusted Odds Ratio
CI: confidence interval
COR: crudes odds ratio
CVD: cardiovascular disease
FT3: free triiodothyronine
FT4: free thyroxine
GABA: gamma amino‐butyric acid
HIV/AIDS: human immunodeficiency virus/acquired immune deficiency syndrome
MCI: mild cognitive impairment
MDD: major depressive disorder
mIU/L: milli international unit per liter
MMAS: Morisky medication‐adherence scale
MMSES: mini‐mental state examination scoring
MoCA: Montreal cognitive assessment
OCD: obsessive‐compulsive disorder
OPD: outpatient department
PHQ‐9: patient health questionnaire‐9
pmol/L: picomole/liter
PSQI: Pittsburg sleep quality index
**ROS**‐: reactive oxygen species
TSH: thyroid‐stimulating hormone
VIF: variance inflation factor

## 1 Introduction

Cognitive impairment is an inclusive term used to describe the impairment of different domains of cognition [1]. It refers to the decline in cognitive functions such as memory, attention, and executive function [2]. With over 55 million people worldwide living with dementia, the growing prevalence of these conditions has profound physical, emotional, and financial impacts [3].

One area of growing concern is the link between hyperthyroidism and cognitive health [4]. Hyperthyroidism can cause difficulties with memory, focus, decision‐making, and even mood disorders [5, 6, 7]. High levels of free thyroxine (FT4) and low levels of thyroid‐stimulating hormone (TSH) increased the risk of cognitive decline and even dementia [8, 9].

There are several mechanisms for how cognitive impairments are seen in hyperthyroid patients. Hyperthyroidism was linked to increased neuroinflammation and necroptosis [10], causing excitotoxicity and oxidative stress [11, 12], an increase in reactive oxygen species (ROS), molecules that harm cellular structures, leading to neuron death and cognitive dysfunction [13, 14]. Additionally, chronic hyperthyroidism can activate microglia, the brain's immune cells, leading to neuroinflammation. This inflammation disrupts communication between neurons and impairs brain plasticity [15]. Hyperthyroidism also raises the risk of cerebrovascular problems such as stroke and conditions like hypertension and atrial fibrillation, resulting in vascular cognitive impairment [16]. Moreover, thyroid hormones play a role in the production and regulation of neurotransmitters such as serotonin, dopamine, and gamma‐aminobutyric acid (GABA). Disruption of these neurotransmitters can have negative impacts on behavior, mood, and thought processes [17, 18].

Determining the etiologies of cognitive impairment is very difficult [19]. But here are some risk factors that contribute to the occurrence of cognitive impairment. Socio‐demographic risk factors are increasing age [20, 21, 22], whereas some studies say younger adults [21], lower educational levels [22], and female sex [9, 22, 23]. Medical risk factors are cardiovascular diseases (CVD), hypertension, high cholesterol, diabetes mellitus, stroke [19], advanced HIV/AIDS patients [24], anemia, being overweight or obese [3], and thyroid diseases [22, 25, 26]. Psychological risk factors are depression [27], post‐traumatic stress disorder [28], and anxiety [29]. Lifestyle and behavioral risk factors are smoking [30], khat chewing [24], heavy alcohol consumption [31], lack of physical exercise [22, 32, 33], and being socially isolated [3]. Head injury, genetic susceptibility [19], and increased duration of hyperthyroidism [8] are also risk factors for cognitive impairment.

Hyperthyroidism affects all populations worldwide and has the potential to have catastrophic health effects [34]. While this connection has been explored in other parts of the world, little is known about how hyperthyroidism affects brain function in Ethiopia. This lack of data is especially concerning as cognitive disorders are on the rise globally [2], and understanding their local drivers is critical for effective healthcare strategies. This study aims to explore the prevalence of cognitive impairment among adults with hyperthyroidism and identify the factors that associate to it.

## 2 Materials and Methods

### 2.1 Study Area and Period

We conducted the study in hospitals across the Bale Zone, Oromia Region. The hospitals included Goba Referral Hospital, Dello‐Mena General Hospital, Robe General Hospital, and Goro Primary Hospital. Our study period between February 1, 2024, and May 30, 2024.

### 2.2 Study Design

An institution‐based cross‐sectional study design was employed among hyperthyroid patients attending Bale Zone Hospitals, Southeast Ethiopia.

### 2.3 Population

#### 2.3.1 Source Population

All hyperthyroid patients attended Bale Zone Public Hospitals.

#### 2.3.2 Study Population

All hyperthyroid patients who attended treatment at Bale Zone Public Hospitals during the data collection period were included in our study.

### 2.4 Inclusion and Exclusion Criteria

#### 2.4.1 Inclusion Criteria

All already‐diagnosed hyperthyroid adults (≥ 18 years) who are on appropriate therapy and newly diagnosed were included in the study.

#### 2.4.2 Exclusion Criteria

Patients who had a history of seizure, epilepsy, stroke, critical illness, a hearing problem, a vision problem, or known severe psychiatric illnesses (such as schizophrenia, major depressive disorder (MDD), bipolar disorder, and obsessive‐compulsive disorder (OCD)) were excluded.

### 2.5 Sample Size Determination

The actual sample size was determined by using the single population proportion formula. Since there is no similar study conducted in Ethiopia or other African countries, the following assumptions were taken: 95% confidence interval (*Z*_*α*/2_ = 1.96), 50% proportion (*p*), and 5% margin of error (*d*).

$$n=\frac{{\left(Z_{\alpha /2}\right)}^{2}p\left(q\right)}{d^{2}},$$

where *Z* statistics (at 95% CI = 1.96), *p* (population proportion), and *d* (margin of error) $n=\frac{{\left(1.96\right)}^{2}0.5*0.5}{{\left(0.05\right)}^{2}}=384$ with a 10% non‐response rate, it was 423.

### 2.6 Sampling Technique

A sampling frame was prepared for the total number of hospitals in Bale Zone. Then each study participant was selected using a systematic random sampling method. The proportional allocation of patients in each hospital summarized in Table 1 below.

**Table 1:** Proportional allocation of study participants to each hospital (*n* = 423).

S/N	Name of hospital	Previous year (2022/2023) report	Proportional allocation for current study
1	Goba referral hospital	262	118
2	Dello‐Mena general hospital	224	101
3	Robe general hospital	245	110
4	Goro primary hospital	209	94
Grand total		940	423

### 2.7 Data Collection Tools/Instruments

We use a structured interviewer‐administered questionnaire. The other tools were the Mini‐Mental State Examination scoring (MMSES) tool for cognitive impairment, the Pittsburgh Sleep Quality Index (PSQI) tool for sleep quality, the Wealth index tool, and the Morisky Medication‐Adherence Scale (MMAS).

### 2.8 Data Collection Procedure

Data collectors were recruited and trained to collect the data for this study. The data was gathered through face‐to‐face interviews using a questionnaire, and additional information was reviewed from patient registries and medical charts.

Hyperthyroid patients who met the study criteria were recruited during their visits to the surgical outpatient department (OPD) for diagnosis or follow‐up appointments. The data collection process had two parts. In the first part, an interviewer‐administered questionnaire was used to gather information about the patients' sociodemographic details, clinical background, history of substance use, and factors linked to cognitive impairment. At the time of enrollment, the medical history was obtained, and serum levels of FT4, TSH, and FT3 were measured. In the second part, the interviewers assessed the cognitive status of the hyperthyroid patients using educationally adjusted MMSES tool.

### 2.9 Study Variables

#### 2.9.1 Dependent Variable

Cognitive impairment.

#### 2.9.2 Independent Variables

• Socio‐demographic variables: age, sex, marital status, residence, educational level, occupation, and family monthly income
• Behavioral factors: smoking, khat chewing, alcohol consumption, and exercise
• Thyroid function tests: TSH, T4, and T3 levels
• Comorbid medical conditions: CVD, hypertension, diabetes mellitus, high cholesterol, stroke, advanced HIV/AIDS, anemia, depression, and thyroid diseases
• Duration of illness
• Hyperthyroid medication adherence
• Sleep quality

### 2.10 Operational Definition

Cognitive function impairment: Based on the score of the Mini‐Mental State Examination, it was classified as follows [35]:

- No cognitive function impairment if the score is 24–30.
- Cognitive function impairment, if the score is 0–23

Hyperthyroidism: If serum TSH is low (< 0.45 mIU/L) and FT4 > 24.5 pmol/L or FT3 > 6.3 pmol/L or both [36].

Mini‐mental state examination scoring (MMSES) tool: The total score is out of 30, and a generally normal score is ≥ 24 points [35]. But as per a validation study done in Ethiopia, different cut‐offs were used depending on educational level: 22, 24, and 26 for no or primary level education points for those < 8 years of education; for secondary level education (9–12 years of education) and college level and above (≥ 13 years of education), respectively [37]. We have used the educationally adjusted criteria to decide whether there is cognitive impairment or not.

Dementia: a disabling syndrome characterized by a decline in memory and cognition [38].

Pittsburgh Sleep Quality Index (PSQI): A self‐rated questionnaire of sleep disruptions and quality over a period of 1 month. Seven “component” scores—subjective sleep quality, sleep latency, length, habitual sleep efficiency, sleep disruptions, use of sleeping medication, and daytime dysfunction—are produced from 19 separate items. One global score is produced by adding the scores for these seven components [39].

Depression: A mental disorder marked by persistent sadness, discouragement, loss of self‐worth, and loss of interest in usual activities, and with PHQ‐9 score of 5, 10, 15, and 20 representing mild, moderate, moderately severe, and severe depression, respectively [40].

Morisky Medication‐Adherence Scale (MMAS): A four‐question survey evaluates self‐reported medication‐taking behavior [41].

### 2.11 Statistical Analysis

Once the data was collected, it was reviewed for completeness, clarity, and consistency. It was then entered and cleaned using Epidata Manager 4.6.0.0 before being analyzed with Stata 17. The analysis began with descriptive statistics, followed by binary logistic regression. The findings were presented in the form of text, graphs, and tables. To explore the relationship between independent variables and outcomes, variables with a *p* value less than 0.25 in pairwise analysis were included in the multiple‐variable logistic regression model. The Hosmer–Lemeshow test was used to check how well the model fit the data, yielding a result of 0.109. Additionally, multicollinearity among explanatory variables was evaluated using the variance inflation factor (VIF), which had a value of 2.12, which is within the acceptable range of 1–5. Finally, variables with an odds ratio, a 95% confidence interval, and analyses that were two‐tailed with a *p* value below 0.05 were identified as statistically significant.

### 2.12 Data Quality Control

Before the data collection began, the questionnaire was tested to ensure its accuracy and consistency. Data collectors were trained on the study's objectives, its importance, participant confidentiality, rights of participants, obtaining consent, effective interview techniques, and maintaining quality control throughout the process.

To prevent sampling bias, a sample size large enough was calculated to address all study objectives. The questionnaire was translated into Afan Oromo and Amharic to ensure clarity and inclusivity. A pre‐test was then conducted with 5% of the sample size. Supervisors were assigned to oversee the process daily, ensuring everything was conducted smoothly and according to plan.

### 2.13 Ethical Consideration

Ethical clearance and support letters were obtained from the ethical review board of Madda Walabu University, referenced as Rmu‐17/122/383. Additionally, permission to conduct the survey was granted by the relevant authorities of each hospital and their respective departments. Both written and verbal consent were obtained from each participant before data collection began. To protect participants' privacy and ensure confidentiality, their names were not included on the questionnaire, and unique codes were used. Any abnormal findings were shared with the participants, and they were referred to their physicians for follow‐up care.

## 3 Results

A total of 406 (response rate of 96%) hyperthyroid patients participated in the study, with 112 from Goba Referral Hospital, 98 from Dello‐Mena General Hospital, 105 from Robe General Hospital, and 93 from Goro Primary Hospital. The average age of the participants was 41.03 ± 13.89 years, and their ages ranged from 18– 80 years. Most of the participants were female, making up 371 (91%) of the sample, as detailed in Table 2.

**Table 2:** Sociodemographic characteristics of adult hyperthyroid patients who attended Bale Zone Hospitals, Southeast Ethiopia, 2024 (*n* = 406).

Variable	Category	Frequency (%)
Sex	Male	35 (8.6)
	Female	371 (91.4)
Marital status	Single	33 (8.1)
	Married	344 (84.7)
	Widowed	16 (3.9)
	Separated	13 (3.2)
Education status	Grade 8 and lower	263 (64.8)
	Secondary school	106 (26.1)
	Higher education	37 (9.1)
Occupation	Employed	53 (13.1)
	Unemployed	353 (86.9)
Residence	Urban	218 (53.7)
	Rural	188 (46.3)
Wealth index	Poor	163 (40.2)
	Middle	81 (19.9)
	Rich	162 (39.9)

### 3.1 Sociodemographic, Clinical Characteristics, and Substance Use

On average, hyperthyroidism had been diagnosed for 4.38 ± 3.50 years, with the duration varying between 1 and 20 years. The average levels of TSH, FT3, and FT4 showed some variation, with TSH averaging 0.28 ± 0.10 mIU/L, FT3 averaging 26.46 ± 21.02 pmol/L, and FT4 averaging 63.29 ± 40.48 pmol/L.

Among all the participants, 119 individuals (29.3%) had one or more co‐existing conditions. Regarding substance use, only 17 participants (4.2%) reported consuming alcohol in the past month. Additionally, 15 participants (3.7%) had a history of smoking, 13 (3.2%) had previously chewed khat, and 16 (3.9%) were currently chewing. As for physical activity, 41 participants (10.1%) engaged in some form of exercise 1–5 days a week. For sleep quality, 253 (62.3%) have poor sleep quality, and regarding adherence to hyperthyroid medication, 164 (46.7%) are moderate and 160 (45.6%) good, as shown in Table 3.

**Table 3:** Clinical characteristics and substance use history of adult hyperthyroid patients who attended Bale Zone Hospitals, Southeast Ethiopia, 2024 (*n* = 406).

Variable	Category	Frequency (%)
Comorbidity	Yes	119 (29.3)
	No	287 (70.7)
Smoking status	Never smoke	391 (96.3)
	History of smoking	15 (3.7)
Alcohol consumption	Yes	17 (4.2)
	No	389 (95.8)
Khat chewing status	Never chewing	377 (92.9)
	History of chewing	13 (3.2)
	Currently chewing	16 (3.9)
Physical exercise	Yes	41 (10.1)
	No	365 (89.9)
Sleep quality	Good	153 (37.7)
	Poor	253 (62.3)
Drug adherence	Poor	27 (7.7)
	Moderate	164 (46.7)
	Good	160 (45.6)

### 3.2 Prevalence of Cognitive Impairment

**Figure 1:**
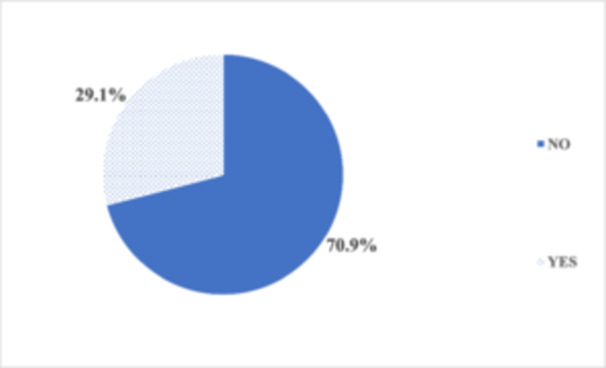
Prevalence of cognitive impairment among adult hyperthyroid patients at Bale Zone hospitals, Southeast Ethiopia, 2024 (*n *= 406).

The prevalence of cognitive impairment was found to be 29.1% (95% CI: 24.84–33.68), as shown in Figure 1. It was more common among females (105 or 89%), those from rural areas (62 or 52.5%), and people with lower education levels (90 or 76.3%). For those with cognitive impairment, the average TSH level was 0.27 mIU/L (95% CI: 0.27–0.29), lower than that of individuals with normal cognitive function. Additionally, the FT4 and FT3 levels in those with cognitive impairment were 89.23 pmol/L (95% CI: 81.05–97.41) and 37.37 pmol/L (95% CI: 32.88–41.87), both of which were higher than in individuals with normal cognitive status.

### 3.3 Factors Associated With Cognitive Impairment

The independent variables that had a *p* value of less than 0.25 in the bivariable logistic regression analysis were included in the multivariable logistic regression analysis. These factors included age, marital status, education level, place of residence, disease duration, history of hypertension, alcohol consumption, comorbidities, FT3 and TSH levels, wealth index, sleep quality, and medication adherence.

The multivariable logistic regression analysis revealed several factors significantly associated with cognitive impairment in hyperthyroid patients. Age (AOR = 1.09, 95% CI [1.06, 1.12]), alcohol consumption (AOR = 0.16, 95% CI [0.04, 0.62]), FT3 levels (AOR = 1.03, 95% CI [1.01, 1.05]), wealth index (middle: AOR = 0.18, 95% CI [0.07, 0.49]; rich: AOR = 0.19, 95% CI [0.07, 0.51]), and sleep quality (AOR = 2.24, 95% CI [1.12, 4.46]) were all significantly linked to hyperthyroid‐associated cognitive impairment. Specifically, the likelihood of cognitive impairment increased by 9% for a year increase in age. Additionally, for every unit increase in FT3 levels, the probability of cognitive impairment rose by 3%. As shown in Table 4, FT4 was significant in binary logistics, but it was removed from the multivariable logistic regression analysis because it affects the model with a large variable inflation factor.

**Table 4:** Factors associated with cognitive impairment among adult hyperthyroid patients in bivariable and multivariable binary logistic regression, Bale Zone Hospitals, Southeast Ethiopia, 2024 (*n* = 406).

Variable		Cognitive impairment		COR (95% CI)	*p* value	AOR (95% CI)	*p* value
		Yes (*n* = 118)	No (*n* = 288)				
		Frequency (%)	Frequency (%)				
Age (years)^a^		50.88 ± 15.34	36.99 ± 10.95	1.08 (1.06, 1.10)	< 0.001	1.09 (1.06, 1.12)	< 0.001*
Educational level	Grade 8 and lower	90 (34.22)	173 (65.78)	2.69 (1.08, 6.68)	0.03	0.55 (0.13, 2.34)	0.42
	Secondary school	22 (20.75)	84 (79.25)	1.35 (0.50, 3.65)	0.55	0.89 (0.21, 3.85)	0.88
	Higher education	6 (16.22)	31 (83.78)	1		1	
Marital status	Single	6 (18.18)	27 (81.82)	1		1	
	Married	246 (71.51)	98 (28.49)	1.79 (0.72, 4.48)	0.21	0.33 (0.09, 1.17)	0.09
	Other^b^	14 (48.28)	15 (51.72)	4.19 (1.34, 13.21)	0.01	0.53 (0.12, 2.54)	0.43
Residence	Urban	56 (25.69)	162 (74.31)	1		1	
	Rural	62 (32.98)	126 (67.02)	1.42 (0.93, 2.19)	0.11	1.09 (0.47, 2.57)	0.84
Disease duration^a^		4.81 ± 3.83	4.21 ± 3.35	1.05 (0.99, 1.11)	0.12	0.94 (0.86, 1.03)	0.16
History of hypertension	No	96 (26.67)	264 (73.33)	1		1	
	Yes	22 (47.83)	24 (52.17)	2.52 (1.35, 4.70)	0.004	1.09 (0.36, 3.37)	0.88
Alcohol consumption	No	109 (28.02)	280 (71.98)	0.35 (0.13 0.92)	0.03	0.16 (0.04, 0.62)	0.009*
	Yes	9 (52.94)	8 (47.06)	1		1	
Comorbidity	No	73 (25.44)	214 (74.56)	1		1	
	Yes	45 (37.82)	74 (62.18)	1.78 (1.13, 2.81)	0.01	1.44 (0.62, 3.34)	0.39
TSH (mIU/L)^a^		0.27 ± 0.08	0.28 ± 0.12	0.28 (0.03, 2.41)	0.25	0.25 (0.01, 6.05)	0.39
FT3 (pmol/L)^a^		37.37 ± 24.82	21.99 ± 17.41	1.04 (1.02, 1.05)	< 0.001	1.03 (1.01, 1.05)	< 0.001*
FT4 (pmol/L)^a^		89.23 ± 45.22	52.66 ± 32.99	1.02 (1.01, 1.03)	< 0.001		
Wealth index	Poor	73 (44.79)	90 (55.21)	1		1	
	Middle	13 (16.05)	68 (83.95)	0.24 (0.12, 0.46)	< 0.001	0.18 (0.07, 0.49)	0.001*
	Rich	32 (19.75)	130 (80.25)	0.30 (0.19, 0.50)	< 0.001	0.19 (0.07, 0.51)	0.001*
Sleep quality	Good	36 (23.53)	117 (76.47)	1		1	
	Poor	82 (32.41)	171 (67.59)	1.56 (0.99, 2.46)	0.06	2.24 (1.12, 4.46)	0.02*
Drug adherences	Poor	12 (44.44)	15 (55.56)	1		1	
	Moderate	57 (34.76)	107 (65.24)	0.67 (0.29, 1.52)	0.33	0.51 (0.15, 1.73)	0.28
	Good	32 (20.00)	128 (80.00)	0.31 (0.13, 0.73)	0.007	0.30 (0.09, 1.06)	0.06

Abbreviations: AOR, adjusted odds ratio; CI, confidence interval; COR, crude odds ratio; FT3, free triiodothyronine; FT4, free thyroxine; mIU/L, milli international unit per liter; pmol/L, picomole/liter.

^a^For continuous variables presented as mean ± standard deviation (SD).

^b^For widowed and separated combined.

*For statistically significant (*p *< 0.05).

## 4 Discussion

This study explored the prevalence of cognitive impairment and its associated factors among adult hyperthyroid patients in hospitals throughout the Bale zone. The findings revealed that 29.1% of adult hyperthyroid patients experienced cognitive impairment (95% CI [24.62, 33.49]). This is consistent with a research result that 29.2% of thyroid patients experience memory loss [42]. Hyperthyroidism raises the risk of developing severe cognitive impairment by 1.14 times [4].

Age was found to be significantly associated with cognitive impairment, with an AOR of 1.09 (95% CI: 1.06–1.12, *p* < 0.001), meaning that older age was linked with cognitive impairment. This finding is consistent with other research, like one that found MCI affects roughly 16% of people aged 70 and older [43]. The decline in cognitive abilities like processing speed and memory often begins in the 30s and becomes more pronounced with age [44, 45]). It becomes more evident as individuals age, particularly after 60 [44, 46]. As people get older, they become more vulnerable to both normal age‐related cognitive decline and pathological conditions like dementia [45]. Moreover, cognitive dysfunction is more prevalent among older hyperthyroid patients, emphasizing the importance of considering thyroid function when evaluating cognitive health in aging populations [47].

Higher levels of FT3 have been associated with cognitive impairment; it has an AOR of 1.03 (95% CI: 1.01, 1.05, *p* < 0.001). This suggests a significant positive relationship between higher FT3 levels and cognitive decline [48, 49]. Moreover, higher FT3 levels are linked to poorer cognitive performance in patients with type 2 diabetes [48]. Thyroid hormones play a key role in neurotransmission and neuronal plasticity, both of which are essential for cognitive function. Disruptions in thyroid hormone levels can lead to cognitive deficits [49, 50]. Impaired sensitivity to thyroid hormones has also been associated with MCI [49]. However, some studies indicate no consistent connection between thyroid dysfunction and cognitive decline, suggesting that the relationship may be more complex and influenced by other factors such as age and comorbidities [23].

Non‐drinkers were significantly less likely to experience cognitive impairment compared to those who consumed alcohol. The AOR was 0.16 (95% CI: 0.04–0.62, *p* = 0.009), highlighting a protective effect against cognitive decline for individuals who abstain from consuming alcohol. Avoiding alcohol consumption is associated with better cognitive outcomes compared to drinking behaviors, especially heavy or binge drinking [51, 52, 53]. For instance, studies have found that problematic alcohol consumption is strongly linked to faster cognitive decline and an increased risk of severe cognitive impairment [54]. While research suggests that moderate alcohol consumption might have cognitive benefits [55]. Alcohol's neurotoxic effects, combined with the excess thyroid hormones seen in hyperthyroidism, may lead to heightened neuronal damage and worsened cognitive function. Cognitive and behavioral impairments are already common in hyperthyroid patients, significantly affecting their quality of life [56]. Alcohol can amplify these issues by exacerbating symptoms like impulsivity and emotional instability [57]. This intensification may lead to risky behaviors, further jeopardizing cognitive health. Hyperthyroidism itself is associated with alterations in brain connectivity, particularly in regions involved in emotion regulation and memory [58, 59]. Alcohol's impact on these same areas could disrupt cognitive processes even more. Research indicates that hyperthyroidism can cause changes in brain structure and function, particularly in areas responsible for executive function and decision‐making [60]. Alcohol consumption is linked to cognitive decline by introducing additional neurotoxic stressors [61].

Although alcohol consumption was significantly associated with the outcome in the multivariable logistic regression analysis (AOR = 0.16, 95% CI: 0.04–0.62), only 17 participants reported alcohol consumption. This small number of alcohol consumers may have reduced the stability and precision of the estimated odds ratio, making the estimate sensitive to small changes in the data. Therefore, the observed association should be interpreted with caution and confirmed in future studies with a larger number of alcohol consumers.

People in the middle and wealthy categories were found to have a much lower risk of cognitive impairment compared to those in the poorer category, as indicated in this research. Specifically, for those in the middle wealth group, the AOR was 0.18 (95% CI: 0.07–0.49, *p* < 0.001), and for the rich group, it was 0.19 (95% CI: 0.07–0.51, *p* < 0.001). This suggests that individuals with higher socioeconomic status tend to experience fewer cognitive problems. Socioeconomic status is a well‐known factor influencing health outcomes, including cognitive function [62]. Those in higher wealth index categories often have better access to healthcare, healthier living conditions, and fewer stressors that could negatively affect brain health [62]. On the other hand, people in lower wealth categories often face higher levels of stress, limited healthcare access, and a greater likelihood of co‐existing health conditions, which can worsen cognitive decline [63]. Additionally, individuals in poorer socioeconomic groups may not receive adequate treatment for hyperthyroidism [64]. Lower socioeconomic status is also linked to lifestyle factors such as poor diet and higher alcohol consumption [62]. Chronic stress, often experienced by those in lower wealth categories, can further worsen the symptoms of hyperthyroidism, compounding cognitive difficulties [63].

Poor sleep quality was strongly linked to a higher likelihood of cognitive impairment. The AOR for poor sleep quality was 2.24 (95% CI: 1.12–4.46, *p* = 0.02). Poor sleep has increasingly been recognized as a major contributor to cognitive decline, affecting attention, memory, and executive function [65, 66, 67]. This impact is even more significant in people with underlying health conditions like thyroid disorders [68]. Hyperthyroidism is often associated with sleep problems like insomnia, longer time to fall asleep, and difficulty staying asleep [68]. As a result, individuals with hyperthyroidism may experience worse sleep quality. The link between poor sleep and cognitive decline is especially crucial for those with hyperthyroidism. Cognitive function can deteriorate faster in people who also suffer from sleep disturbances [69]. Improving sleep quality in those with hyperthyroidism could therefore be key to enhancing cognitive outcomes [70].

### 4.1 Implications of Study

The findings of this study provide the following key practical implications:

Clinical practice: Routine screening for cognitive impairment should be integrated into the care of hyperthyroid patients, especially older adults and those with high FT3 levels or poor sleep.

Public health: Awareness programs are needed to highlight the cognitive effects of thyroid disease and promote early diagnosis and treatment.

Policy: Findings support the need for guidelines that include cognitive assessment as part of hyperthyroidism management.

Research: The study provides a baseline for future longitudinal and interventional studies examining the causal pathways linking thyroid dysfunction with cognitive decline.

## 5 Strengths and Limitations of This Study

The study has the following significant strengths. First, we use a validated and standardized cognitive assessment tool (MMSE with Ethiopian cut‐offs), which increases the reliability of results. Second, a large sample size and multi‐hospital data collection enhance representativeness within the study area. Third, the study accounted for a wide range of socio‐demographic, clinical, behavioral, and biochemical factors, providing a comprehensive analysis of associated factors.

However, the study has the following limitations: first, the study's cross‐sectional nature limits its ability to establish causal relationships between hyperthyroidism and cognitive impairment. Second, the study does not stratify cognitive impairment by the severity of hyperthyroidism and different age groups. Thirdly, laboratory markers which could enhance diagnostic accuracy, such as inflammatory biomarkers or brain imaging, were not included.

## 6 Conclusion and Recommendations

This study found that nearly one‐third of thyroid patients experienced cognitive impairment. It indicated that cognitive impairment is a significant and underrecognized problem in this population. Advanced age, elevated FT3 level, poor sleep quality, and low socioeconomic status were the strongest associated factors of cognitive decline. These findings highlight the importance of routinely assessing cognitive function in hyperthyroid patients and addressing modifiable associated factors such as sleep problems and delayed treatment. Improving thyroid disease management and integrating cognitive screening in routine care may help to reduce the burden of cognitive impairment.

## Author Contributions

**Ephrem Tesfaye Mihretie:** conceptualization, investigation, funding acquisition, writing – original draft, writing – review and editing, supervision, data curation, formal analysis. **Girmaye Tesfaye:** conceptualization, investigation, methodology, validation, software, formal analysis, data curation, writing – original draft, writing – review and editing, visualization. **Yordanos Sintayehu:** conceptualization, investigation, validation, data curation, methodology, formal analysis, software, writing – original draft, writing – review and editing, visualization. **Ferhan Ahmed:** conceptualization, investigation, methodology, validation, software, formal analysis, data curation, writing – original draft, writing – review and editing, visualization. **Bereket Gezahegn:** conceptualization, investigation, methodology, validation, software, formal analysis, data curation, writing – original draft, writing – review and editing, visualization. **Niguse Serbecha:** conceptualization, investigation, methodology, validation, software, formal analysis, data curation, writing – original draft, writing – review and editing, visualization. **Zerihun Shiferaw:** conceptualization, investigation, methodology, validation, software, formal analysis, data curation, writing – original draft, writing – review and editing, visualization. **Awel Turki:** conceptualization, investigation, methodology, validation, software, formal analysis, data curation, writing – original draft, writing – review and editing, visualization. **Fikreab Desta:** conceptualization, resources, data curation, investigation, formal analysis, methodology, project administration, writing – original draft, writing – review and editing, validation.

## Consent

Informed consent was obtained from all individual participants included in the study.

## Conflicts of Interest

The authors declare no conflicts of interest.

## Transparency Statement

The corresponding author, Ephrem Tesfaye Mihretie, affirms that this manuscript is an honest, accurate, and transparent account of the study being reported; that no important aspects of the study have been omitted; and that any discrepancies from the study as planned (and if relevant, registered) have been explained.

## Data Availability

We included all the relevant information; if additional data is required, it can be obtained from the corresponding author if required.
